# Lower respiratory tract microbiome and lung cancer risk prediction in patients with diffuse lung parenchymal lesions

**DOI:** 10.3389/fcimb.2024.1410681

**Published:** 2024-08-09

**Authors:** Xiaochang Wang, Tianchi Xiao, Mingqing Lu, Zhaoqing Wu, Lingdan Chen, Zili Zhang, Wenju Lu

**Affiliations:** ^1^ State Key Laboratory of Respiratory Diseases, National Clinical Research Center for Respiratory Disease, National Center for Respiratory Medicine, Guangzhou Institute of Respiratory Health, The First Affiliated Hospital of Guangzhou Medical University, Guangzhou, Guangdong, China; ^2^ KingMed School of Laboratory Medicine, Guangzhou Medical University, Guangzhou, Guangdong, China

**Keywords:** lung cancer, diffuse lung parenchymal lesions, bronchoalveolar lavage fluid, microbiome, metagenomic next-generation sequencing, biomarker

## Abstract

**Objective:**

In clinical practice, imaging manifestations of diffuse lung parenchymal lesions are common and indicative of various diseases, making differential diagnosis difficult. Some of these lesions are eventually diagnosed as lung cancer.

**Methods:**

Because respiratory microorganisms play an important role in lung cancer development, we searched for microbial markers that could predict the risk of lung cancer by retrospectively analyzing the lower respiratory tract (LRT) microbiome of 158 patients who were hospitalized in the First Affiliated Hospital of Guangzhou Medical University (March 2021–March 2023) with diffuse lung parenchymal lesions. The final diagnosis was lung cancer in 21 cases, lung infection in 93 cases, and other conditions (other than malignancy and infections) in 44 cases. The patient’s clinical characteristics and the results of metagenomic next-generation sequencing of bronchoalveolar lavage fluid (BALF) were analyzed.

**Results:**

Body mass index (BMI) and LRT microbial diversity (Shannon, Simpson, species richness, and Choa1 index) were significantly lower (P< 0.001, respectively) and *Lactobacillus acidophilus* relative abundance in the LRT was significantly higher (P< 0.001) in patients with lung cancer. The relative abundance of *L. acidophilus* in BALF combined with BMI was a good predictor of lung cancer risk (area under the curve = 0.985, accuracy = 98.46%, sensitivity = 95.24%, and specificity = 100.00%; P< 0.001).

**Conclusion:**

Our study showed that an imbalance in the component ratio of the microbial community, diminished microbial diversity, and the presence of specific microbial markers in the LRT predicted lung cancer risk in patients with imaging manifestations of diffuse lung parenchymal lesions.

## Introduction

Diffuse lung parenchymal lesions are typically characterized by the presence of ground-glass shadows, miliary opacities, diffuse patchy shadows, or diffuse nodules in both lungs on imaging, and their causes are complex (Tomassetti et al., 2017). Some common tumors and infectious diseases of the lungs show similar manifestations that are difficult to distinguish in clinical diagnosis and generally require a confirmatory pathological examination. A proportion of Diffuse lung parenchymal lesions are ultimately diagnosed as lung cancer, most of which are of intermediate to advanced stages, and the 5-year survival rate is poor. Early-stage lung cancer has a 10-year survival rate of 92% if surgical resection is possible. However, the prediction and identification of early-stage lung cancer remain challenging (Chinese Anti-Cancer Association et al., 2021). The gold standard for clinical diagnosis of lung cancer is lung biopsy. Although highly accurate, it has major limitations due to its invasive nature and a low patient acceptance rate (Pabst et al., 2023). Computed tomography (CT) and multislice spiral CT also have high diagnostic value, but radiation exposure limits their use (Hughes et al., 2022). Some new serological markers of lung cancer have been recently reported. For example, Tian et al (Tian et al., 2019). reported the significant downregulation of miR-486-5p in the serum of patients with non-small cell lung cancer, and Huang et al (Huang et al., 2021). identified a variety of circRNAs that differed significantly in the peripheral blood of patients with lung cancer, but the sensitivities and specificities of these markers were unsatisfactory.

The human microbiota comprises bacteria, fungi, archaea, protozoa, and viruses, all of which play important roles in physiological processes and diseases. An increasing number of studies have shown that the microbiota influences cancer occurrence and development (Zitvogel et al., 2017). For example, the presence of *Helicobacter pylori* in the upper gastrointestinal tract significantly increases the risk of gastric cancer (Tan and Wong, 2011), and toxins produced by *Fusobacterium nucleatum* in the gut promote colorectal cancer development (Rubinstein et al., 2019). Lung cancer is a common tumor worldwide, and its mortality rate was reported to be the highest in urban areas (Wu et al., 2022). Liu et al (Liu et al., 2018). collected and sequenced bronchial brush samples from cancerous sites and contralateral non-cancerous sites in patients with lung cancer and from healthy controls and found that the α diversity of the microbiome gradually decreased from the healthy to the non-cancerous to the cancerous site. Furthermore, the abundance of *Streptococcus* and *Neisseria* increased, while that of *Staphylococcus* decreased. In contrast, Greathouse et al (Chen et al., 2021). collected and sequenced pathological tissue samples of cancerous sites and adjacent non-cancerous sites from patients with lung cancer and tissue samples of normal lungs from patients who did not have lung cancer, and the results showed that the α diversity of normal lung flora was lower than that of tumor-adjacent or tumor tissue. These discrepancies indicate that the study of the relationship between lung microbiota and lung cancer is still in the preliminary exploratory stage.

Therefore, this study aimed to analyze the LRT microbiome of patients with Diffuse lung parenchymal lesions on lung imaging to search for microbial markers predictive of lung cancer risk and to provide new approaches for the diagnosis and differential diagnosis of lung cancer.

## Materials and methods

### Subjects

We retrospectively analyzed 158 patients who were hospitalized in the First Affiliated Hospital of Guangzhou Medical University from March 2021 to March 2023 and showed imaging manifestations of diffuse parenchymal lung lesions. Of them, 21 were finally diagnosed with lung cancer, 93 with lung infection, and 44 with other conditions (except malignancy and infections). The clinical characteristics of the patients and metagenomic sequencing results of BALF were summarized and analyzed.

**Figure 1:**
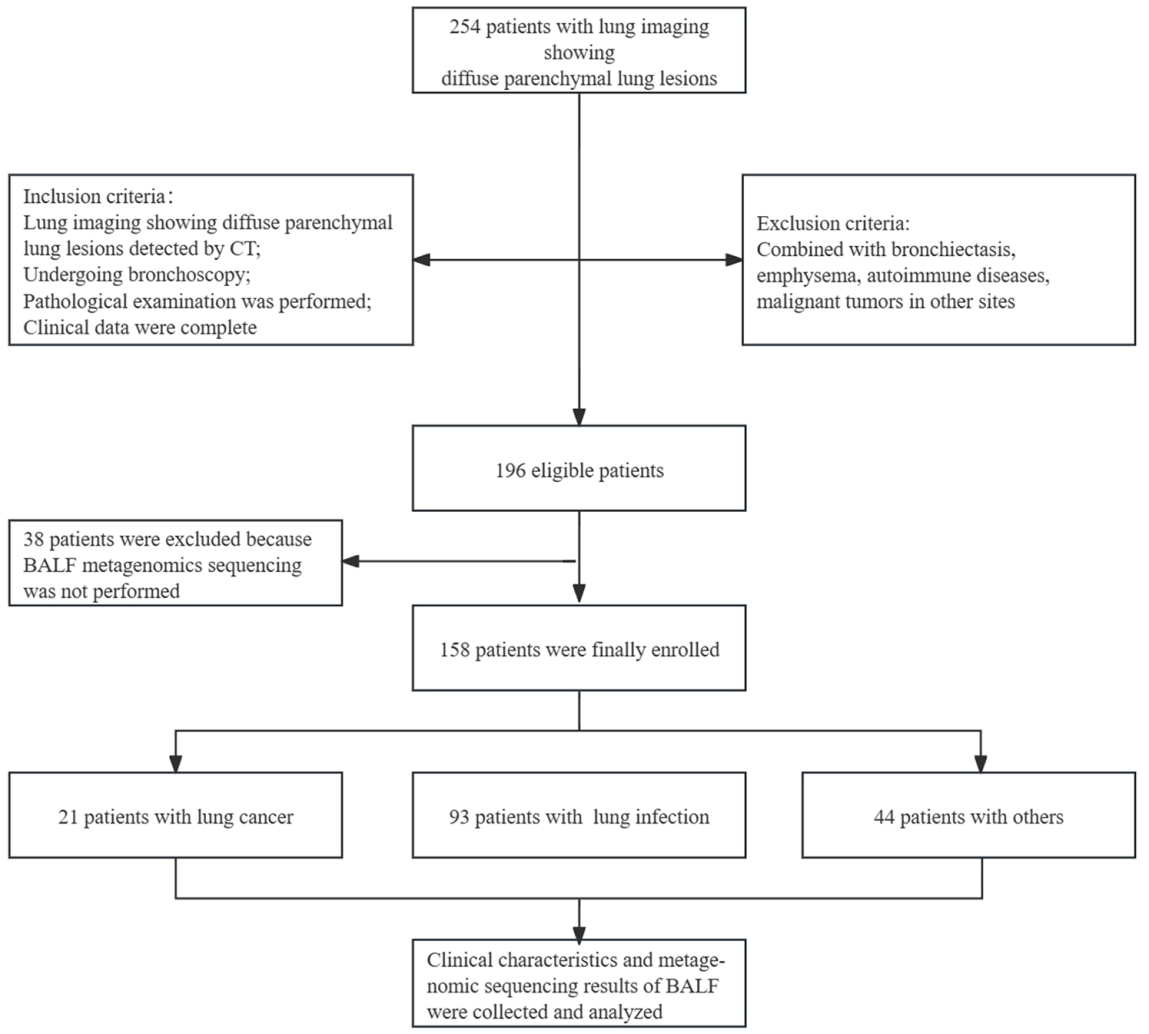
Full inclusion and exclusion criteria, and study design.

This study was registered with the Chinese Clinical Trial Register (www.chictr.org.cn) under registration number ChiCTR-CCC-12002950. All enrolled patients signed informed consent. **Figure 1** presents the study design and the full inclusion and exclusion criteria.

### Bronchoalveolar lavage fluid sampling and processing

A routine clinical status assessment was performed before bronchoalveolar lavage with strict control of indications and contraindications. Briefly, surface anesthesia was performed using nebulized inhaled lidocaine, followed by the injection of 1–2 ml 2% lidocaine into the lavage lung segment through the biopsy hole for local anesthesia. Then, 60 ml room-temperature sterilized saline was rapidly injected through the operated orifices in divided injections. Immediately thereafter, the BALF was collected by suction under negative pressure (<100 mmHg), and the total recovery rate was ≥30%. The collected BALF was divided into two sterile sealed containers for pathogenetic and cytological analysis, respectively (Society, C. R, 2017). Each sample was saved in a 2-6°C cooler, and immediately transported to a -86°C ultra-low-temperature refrigerator for storage until it was thawed before sequencing.

### Nucleic acid extraction and microbiota profiling by metagenomic next-generation sequencing

Nucleic acids were extracted from the BALF, and the DNA library was prepared using the PathoLib™ Genomic Library Construction Kit from Willingmed Technology (Beijing Co., Ltd). The concentrations of nucleic acid, library, and fragment length were measured simultaneously. The library was diluted to the appropriate concentration for sequencing, denatured by adding NaOH to a final concentration of 0.1 nM, mixed with Illumina HT1 solution, and then sequenced on an Illumina NextSeq 550 System (Eisenstein, 2015). Negative controls, positive controls, and internal controls were set up during the aforementioned steps. All reagents were provided by Willingmed Technology (Beijing Co., Ltd).

### Relative quantitative polymerase chain reaction

Relative quantitative PCR assay using the SYBR method. Amplification/oligonucleotide primer pairs were designed for *Lactobacillus acidophilus* based on the characteristics in the nucleotide sequences of the 16S-23S rRNA spacer regions (Forward primer 5’-TCTAAGGAAGCGAAGGAT-3’, Reverse primer 5’-CTCTTCTCGGTCGCTCTA-3’) (Tilsala-Timisjärvi and Alatossava, 1997). 18sRNA was used as an internal reference gene (Forward primer 5’-GCAATTATTCCCCATGAACG-3’, Reverse primer 5’-GGCCTCACTAAACCATCC-3’). PCR reaction conditions: 95°C for 1 minute; 95°C for 20 seconds, 56°C for 1 minute, for a total of 40 cycles. DNA levels were calculated using the 2-^ΔΔCT^ method.

### Bioinformatics analysis

FastQC v0.12.1 (de Sena Brandine and Smith, 2021) was used for quality assessment, and fastp v0.23.4 for quality control of sequencing data. Trimmomatic (Bolger et al., 2014) was used to remove primers, connectors, and low-quality sequences. Host sequences were removed using Kneaddata v0.12.0 (http://huttenhower.sph.harvard.edu/kneaddata) and Bowtie2 v2.5.1 (Langmead and Salzberg, 2012), and the resultant non-host sequences underwent downstream analysis. MetaPhlAn2 (Truong et al., 2015) was used to analyze the composition of the microbiome community and HUMAnN 2 (http://www.huttenhower.org/humann2) to obtain the species abundance and metabolic pathway function information of the microbiome. The above analysis was performed using the EasyMetagenome pipeline (https://github.com/YongxinLiu/EasyMetagenome). The cooccurrence network of the microbiota was generated using Cytoscape v3.7.0 and visualized in a circular layout (Sam Ma et al., 2015).

### Statistical analyses

Data were shown as the mean (standard deviation) or the median (interquartile range) for continuous variables and number (%) for categorical variables. Continuous variables were compared between groups by the one-way ANOVA or Kruskal–Wallis test, and categorical variables were analyzed using the Pearson Chi-square test or Fisher exact test. A two-sided P value< 0.05 was considered statistically significant. Statistical analyses were performed using SPSS Version 22 (IBM, Corp., Armonk, NY, USA) statistical software. Box plots were drawn using GraphPad Prism v9.5 (GraphPad Software, Inc., La Jolla, CA, USA). Principal co-ordinates analysis (PCoA) Plot-Adonis were drawn for the three groups using the Bray-Curtis distance. MedCalc Statistical Software v19.0.4(MedCalc Software bvba, Ostend, Belgium; https://www.medcalc.org; 2019) was used to analyze and compare the receiver operating characteristic (ROC) curves. A microbiome cooccurrence network was established using SparCC (Sparse Correlations for Compositional data) (von Mering et al., 2012) and visualized using Cytoscape software (v3.9.0, National Institute of General Medical Sciences, Bethesda, MD, USA) (Shannon et al., 2003).

## Results

### Characteristics of the study participants

The study cohort comprised 158 patients (average age 62.76 years; 98 males and 60 females). The final diagnoses included 21 cases of lung cancer, 93 cases of lung infection, and 44 cases where the diagnosis was neither malignancy nor infection (others). Of the 21 patients with lung cancer, 16 had adenocarcinomas, 4 had squamous cell carcinomas, and 1 had small cell carcinoma. The clinical staging of these patients was as follows: 5 patients at stage I, 8 patients at stage II, 7 patients at stage III, and 1 patient at stage IV. Body mass index (BMI) was significantly lower in patients with lung cancer compared with patients with lung infection and other diagnoses, and peripheral blood leukocyte counts, and neutrophil percentages were significantly higher in patients with lung infection compared with the other two groups. The characteristics of the study participants and all P values are presented in **Tables 1** and **2**.

**Table 1:** Clinical characteristics of patients.

Variables	Lung cancer N=21	Lung infection N=93	Others N=44	*P* value
Demographic characteristics				
Age, year	62.95±6.91	62.69±12.36	62.64±7.22	0.862^a^
Sex ,M/F	13/8(61.9/38.1)	56/37(60.2/39.8)	29/15(65.9/34.1)	0.814^c^
BMI, kg/m^2^	19.83±1.41	21.82±1.51	22.67±1.82	<0.001^b^
Smoking status,%	11(52.38)	32(34.41)	15(34.09)	0.278^c^
Peripheral Blood				
WBCs,10^9^/L	6.72±1.97	10.26±4.60	6.98±3.11	<0.001^a^
Neu,%	57.76±9.59	74.81±13.08	60.83±10.41	<0.001^a^
Lym,%	35.16±10.07	23.01±13.05	31.96±10.49	<0.001^a^
NLR	1.61(1.26,2.28)	3.67(1.97,6.49)	1.88(1.29,2.58)	<0.001^a^
Histology	AC/SCC/SCLC 16/4/1			
Stage	I/II/III/IV 5/8/7/1			

a P values were obtained by the Kruskal-Wallis test.

b P values were obtained by the One-way ANOVA test.

c P values were obtained by the Pearson Chi-Square test.

BMI, body mass index; WBC, White blood cells; Neu, Neutrophils; Lym, Lymphocytes; NLR, The ratio of neutrophil count to lymphocyte count; AC, Adenocarcinoma; SCC, Squamous cell carcinoma; SCLC, Small cell carcinoma.

**Table 2:** *P* value table for pairwise comparison of the indexes with statistically significant differences in the three groups.

indexes	Others vs. Lung cancer	Others vs. Lung infection	Lung cancer vs. Lung infection
BMI	<0.001	0.012	<0.001
WBCs,	1.000	0.001	<0.001
Neu	1.000	<0.001	<0.001
Lym	1.000	<0.001	<0.001
NLR	1.000	<0.001	<0.001
Richness	<0.001	<0.001	0.003
Shannon	<0.001	<0.001	0.002
Simpson	<0.001	<0.001	0.001
Chao1	<0.001	<0.001	0.026

P values have been adjusted by the Bonferroni for multiple tests.

Metagenomic sequencing of BALF from the 158 patients yielded 310,149,065 sequences, of which 287,595,289 (92.73%) were classified. The average number of sequences per sample was 1,962,968, including 82.13% human, 3.9% microbial, 4.7% low quality, 2.0% duplicates, and 7.27% unclassified.

### The component ratio of LRT microbiota taxa in patients with lung cancer is imbalanced and tends to be homogeneous

**Figure 2:**
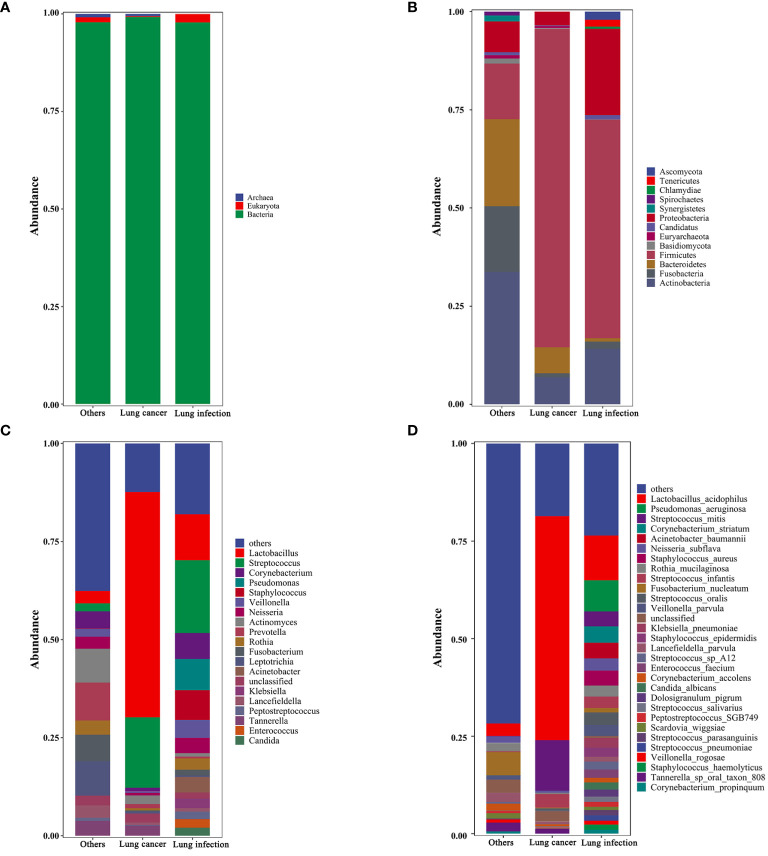
Taxonomic profiles of the LRT microbiota in patients with lung cancer, lung infection, and others. **(A)** Kingdom-level taxonomic profiles. **(B)** Phylum-level taxonomic profiles. **(C)** Genus-level taxonomic profiles. **(D)** Species-level taxonomic profiles. Each vertical bar represents a unique group. The y-axis shows the relative abundance of each taxon. Only the most common taxa are shown. These bar plots were performed on the Tutools platform (https://www.cloudtutu.com), a free online data analysis website. .

Taxonomic analyses of relative abundance were conducted at the kingdom, phylum, genus, and species levels. At the kingdom level, Bacteria were dominant in all study subjects. Eukaryota were present in a smaller proportion in the lung infection and the others groups, but almost absent in the lung cancer group (**Figure 2A**). At the phylum level, Firmicutes, Actinobacteria, Fusobacteria, Bacteroidetes, and Proteobacteria were present in high abundance in all subjects (**Figure 2B**). At the genus level, the lung cancer group was dominated by *Lactobacillus* and *Streptococcus* (both belonging to Firmicutes), while the abundance of all other genera was lower. In the lung infection group, the genera with higher abundance were *Lactobacillus*, *Streptococcus*, *Corynebacterium*, *Pseudomonas*, *Staphylococcus*, *Veillonella*, *Neisseria*, *Acinetobacter*, and *Klebsiella*. In the others group, *Actinomyces*, *Prevotella*, *Fusobacterium*, *Leptotrichia*, *Corynebacterium*, and *Rothia* were in high abundance, and the proportion of each genus was relatively balanced (**Figure 2C**). At the species level, the lung cancer group was dominated by *Lactobacillus acidophilus* and *Streptococcus mitis*. In the lung infection group, common pathogenic bacteria included *Pseudomonas aeruginosa*, *S. oralis*, *Acinetobacter baumannii*, *Staphylococcus aureus*, *Corynebacterium accolens*, *Enterococcus faecium*, *Candida albicans*, and *S. salivarius*. In contrast, the composition of microbial species in the others group maintained its genus-level characteristics and was more balanced, with a relatively high proportion of *F. nucleatum* (**Figure 2D**). Thus, the component ratio of LRT microbiota taxa in patients with lung cancer was imbalanced and tended to be homogeneous. **Table 3** shows the relative abundance of microbial taxa. Heat maps (**Supplementary Figure 1**) were presented to demonstrate the microbiome composition of each sample.

**Table 3:** The microbial taxonomic identification in BALF (Relative abundance).

Microbial taxa	Lung cancer	Lung infection	Others
Kingdom			
Bacteria	99.43±2.57	97.81±11.02	98.08±2.08
Eukaryota	0.20±0.88	2.19±11.02	1.28±1.52
Archaea	0.55±2.45	0.01±0.04	0.85±1.62
Phylum			
*Firmicutes*	81.25±14.56	55.65±36.17	14.21±13.44
*Actinobacteria*	6.92±6.22	14.13±21.75	33.85±8.68
*Proteobacteria*	3.51±3.84	21.93±35.30	7.88±5.25
*Bacteroidetes*	6.59±8.35	0.83±2.92	22.19±9.70
*Fusobacteria*	1.02±2.43	1.85±9.99	16.77±8.40
*Ascomycota*	0	2.1±11.01	0
*Candidatus*	0.16±0.36	1.09±4.78	0.74±0.81
*Basidiomycota*	0.2±0.88	0.09±0.61	1.28±1.52
*Synergistetes*	0.01±0.02	0.01±0.03	1.50±1.89
*Euryarchaeota*	0.55±2.45	0.01±0.04	0.85±1.62
*Spirochaetes*	0.01±0.02	0	1.03±1.70
Genus (Top 20)			
*Lactobacillus*	57.55±27.11	11.66±21.35	3.20±11.78
*Corynebacterium*	0.96±1.93	6.62±18.87	4.43±3.28
*Actinomyces*	2.18±2.88	0.87±2.88	8.72±4.12
*Prevotella*	1.10±2.09	0.47±1.68	9.68±5.77
*Leptotrichia*	0.54±1.67	0.38±0.96	8.83±4.61
*Fusobacterium*	0.21±0.91	1.45±9.94	6.79±4.92
*Pseudomonas*	0	7.97±24.39	0
*Neisseria*	0.65±1.57	3.94±12.98	3.10±3.06
*Staphylococcus*	0.01±0.02	7.59±21.70	0.09±0.13
*Rothia*	0.57±1.19	2.87±6.10	3.66±3.96
*Veillonella*	0.36±0.68	4.54±10.78	1.99±2.62
*Tannerella*	2.59±6.06	0.06±0.23	3.84±3.24
*unclassified*	2.32±3.03	1.54±5.31	2.55±2.64
*Lancefieldella*	0.59±1.24	0.83±2.54	3.15±2.21
*Acinetobacter*	0	3.98±16.72	0
*Peptostreptococcus*	0.20±0.46	1.89±4.99	0.76±1.07
*Enterococcus*	0	2.15±10.87	0
Candida	0	2.07±11.01	0
Species (Top 30)			
*Lactobacillus. acidophilus*	57.55±27.11	11.42±21.40	3.20±11.78
*Pseudomonas. aeruginosa*	0	7.97±24.39	0
*Fusobacterium. nucleatum*	0.18±0.79	1.13±9.90	5.92±4.97
*unclassified*	2.66±3.42	0.51±3.64	3.42±3.02
*Neisseria. subflava*	0.53±1.51	3.13±12.59	1.72±1.92
*Rothia. mucilaginosa*	0.32±0.82	2.77±6.09	2.15±3.23
*Corynebacterium. striatum*	0	4.18±17.01	0
*Lancefieldella. parvula*	0.58±1.23	1.23±3.12	2.24±1.69
*Streptococcus. oralis*	0.64±2.07	3.24±12.21	0.15±0.35
*Acinetobacter. baumannii*	0	3.98±16.72	0
*Veillonella. parvula*	0.09±0.26	2.77±9.67	1.01±1.29
*Staphylococcus. aureus*	0	3.85±16.49	0
*Tannerella_Sp_oral_taxon_808*	1.31±2.81	0	2.27±2.50
*Corynebacterium. accolens*	0.54±1.45	1.19±5.74	1.77±2.84
*Streptococcus_sp_A12*	0.08±0.27	2.01±6.93	0.43±1.10
*Staphylococcus. epidermidis*	0.01±0.02	2.35±11.82	0.09±0.13
*Scardovia. wiggsiae*	0.11±0.35	0.86±6.27	1.37±0.99
*Enterococcus. faecium*	0	2.12±10.87	0
*Peptostreptococcus. SGB749*	0.16±0.45	1.22±3.10	0.55±0.94
*Candida. albicans*	0	1.84±10.87	0
*Veillonella. rogosae*	0.02±0.02	0.99±3.70	0.82±1.81
*Streptococcus. salivarius*	0.30±0.92	1.38±7.89	0.11±0.19
*Streptococcus. parasanguinis*	0.02±0.03	1.33±4.97	0.26±0.90
*Corynebacterium. propinquum*	0	1.00±5.17	0.58±1.18

Only species that were statistically different among the three groups are shown for clarity.

### The LRT microbiome of patients with lung cancer shows a decreased number of species and diminished microbial diversity

**Figure 3:**
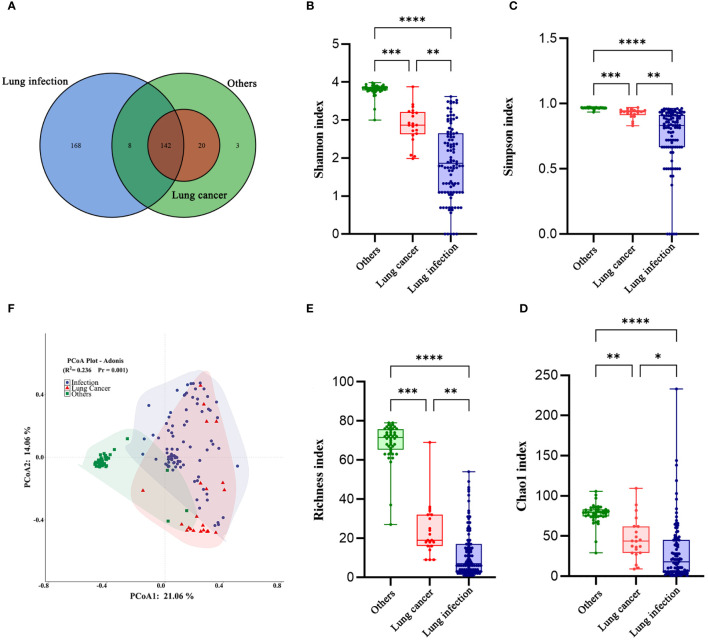
The diversity of the LRT microbiome in patients with lung cancer has changed. **(A)** Venn diagram of the three groups. **(B)** Shannon index, **(C)** Simpson index, **(D)** Choa1 index, and **(E)** Richness index of the LRT microbiome in the three groups. The Kruskal-Wallis test was used, and all *P* values were less than 0.05. Graphpad Pism9.5 software was used for plotting. **(F)** Principal co-ordinates analysis (PCoA) Plot-Adonis based on Bray-Curtis distance for samples in three groups at the genus level. *P<0.05,**P<0.01 ***P<0.001. ****P<0.0001.

We identified 341 types of bacteria at the species level (lung cancer group, 162; lung infection group, 318; and others group, 173). Of them, 168 (49.27%) distinct species were identified in the lung infection group, 3 (0.88%) in the others group, and none in the lung cancer group (**Figure 3A**).

Next, we compared the diversity across different groups of BALF microbiomes. Alpha diversity was lower in the lung cancer group and lung infection group at the genus level (Shannon, Simpson, Choa1, and richness index: all P< 0.001; **Figures 3B–E**), detailed data are presented in **Table 4**. The results of Principal co-ordinates analysis (PCoA) showed significant differences in the composition of the bacterial flora among the three groups (**Figure 3F**). These findings indicated a decreased number of species and diminished microbial diversity in the LRT microbiome of patients with lung cancer.

**Table 4:** The microbiome α diversity indexes in BALF at the genus level.

indexes	Lung cancer	Lung infection	Others	*P* value
Richness	19.00(16.00,32.00)	7.00(3.00,17.00)	71.50(65.25,75.75)	<0.001
Shannon	2.87(2.62,3.21)	1.86(1.10,2.65)	3.84(3.78,3.87)	<0.001
Simpson	0.94(0.91,0.95)	0.83(0.67,0.91)	0.97(0.96,0.97)	<0.001
Chao1	43.60(29.10,62.07)	17.75 (5.50,45.00)	79.35(74.42,83.18)	<0.001

P values were obtained by the Kruskal-Wallis test.

### Microbial biomarkers are enriched in the LRT of patients with lung cancer

**Figure 4:**
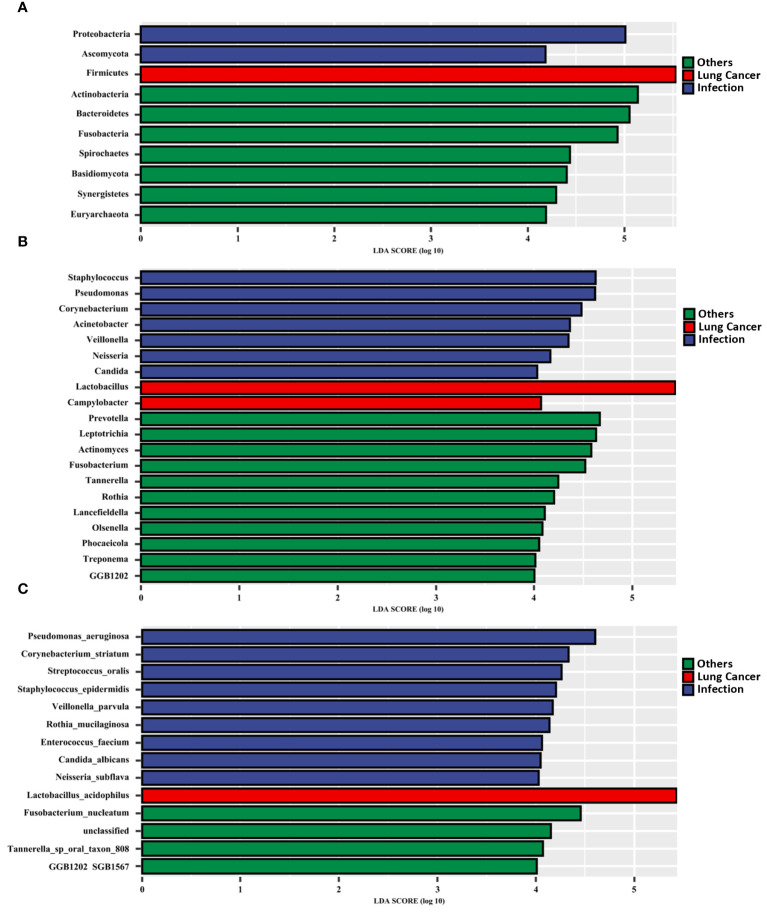
Microbial biomarkers enriched in the LRT of patients with lung cancer. Linear discriminant analysis effect size (LEfSe) revealed different microbiome taxa among all phyla **(A)**, genera **(B)**, and species**(C)** in each group (taxa with LDA score > 4). The LEfSe was performed on the Tutools platform (https://www.cloudtutu.com), a free online data analysis website.

We searched for LRT microbial markers in patients with lung cancer by analyzing differences among all phyla, genera, and species in terms of relative abundance using the linear discriminate analysis effect size (LEfSe). The results showed that specific taxa were enriched in each group (**Figures 4A–C**). Firmicutes, *Lactobacillus*, and *L. acidophilus* were significantly enriched in the lung cancer group. In the lung infection group, *P. aeruginosa*, *C. striatum, S. oralis, S.epidermidis*, *C. albicans*, *E. faecium*, and other common pathogenic bacteria were significantly enriched. In the others group, *Actinomyces*, *Prevotella*, *Fusobacterium*, *Leptotrichia*, *Rothia*, and so on were significantly enriched.

**Figure 5:**
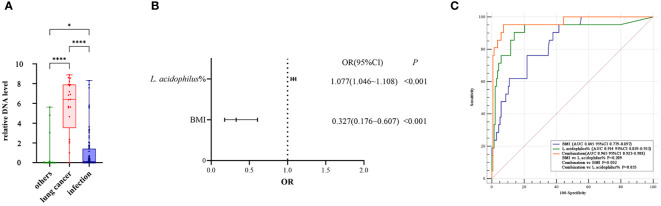
**(A)** The relative DNA levels of *L. acidophilus* in the BALF of patients with lung cancer were significantly higher than that of the other two groups. P values were obtained by Dunn's multiple comparisons test. Graphpad Pism9.5 software was used for plotting. *P<0.05, ****P<0.0001. **(B)** The abundance of *L. acidophilus* in BALF and BMI were used as candidate indices for lung cancer risk prediction. Multivariate logistic regression analyses to establish prediction equation. **(C)** The predictive performance of the model was evaluated by the receiver operating characteristic (ROC) curve. The combination of the two indices showed a higher ROC value than each alone(**Figure 4E**). MedCalc 20.1.4 was utilized to analyze and compare the ROC curves.

The results of the relative quantitative PCR assay showed that the relative DNA levels of *L.acidophilus* in the BALF of patients with lung cancer were significantly higher than those in the other two groups, as shown in **Figure 5A**. The DNA levels of each group and the p-values for two-by-two comparisons are shown in **Table 5**. This indicates the enrichment of microbial biomarkers in the LRT of patients with lung cancer. **Table 3** shows the exact values of the abundance of microbial biomarkers.

**Table 5:** The relative DNA level of *L.acidophilus* amplified by qPCR in BALF.

indexes	Lung cancer	Lung infection	Others	*P* value		
				lung cancer vs. infection	lung cancer vs. others	infection vs. others
relative DNA level	5.75±2.71	0.98±1.81	0.32±1.18	<0.001	<0.001	0.039

P values were obtained by Dunn's multiple comparisons test.

### A combined analysis of the relative abundance of *L. acidophilus* in BALF and BMI provided a better prediction of lung cancer risk

Our dataset was used to establish a diagnostic model for predicting lung cancer risk. The abundance of *L. acidophilus* in BALF and BMI were used as candidate indices for lung cancer risk prediction and were included in multivariate logistic regression analyses to establish prediction equations (P< 0.05, respectively; **Figure 5B**). **Table 6** presents detailed information on the equations.


$$\text{P}=\frac{1}{1+e^{-(19.25\;-\;1.118\;\times \text{ BMI }+\;0.074\;\times \;L.\;acidophilus\text{ \%})}}$$

**Table 6:** The result of multivariate logistic regression analysis.

Variable	B	S.E.	Wald	P value	OR	95% CI	
						Lower	Upper
BMI	-1.118	0.316	12.511	<0.001	0.327	0.176	0.607
*L. acidophilus*%	0.074	0.015	24.552	<0.001	1.077	1.046	1.108
Constant	19.253	6.203	9.614	0.002			

The predictive performance of the model was evaluated according to the area under the curve (AUC). The ROC value was 0.845 (P< 0.001) for BMI and 0.914 (P< 0.001) for *L. acidophilus* %. The combination of the two indices showed a higher ROC value than each one alone (AUC = 0.965, accuracy = 93.04%, sensitivity = 95.24%, and specificity = 92.70%; P< 0.001; **Figure 5C**). We also found a statistically significant difference in AUC between *L. acidophilus* % or BMI alone compared with their combination (P< 0.001, respectively). **Table 7** shows the ROC values.

**Table 7:** The diagnostic value of BMI, *L. acidophilus*% and their combination in predicting the risk of lung cancer.

Variable	Associated criterion	AUC	95% CI		Accuracy	Sensitivity	Specificity	P value
			Lower	Upper				
BMI	≤20.55	0.845	0.779	0.897	77.85	76.19	78.10	<0.001
*L. acidophilus*%	>17.89	0.914	0.859	0.953	86.71	90.48	86.13	<0.001
Combination	>0.12	0.965	0.923	0.988	93.04	95.24	92.70	<0.001
BMI vs *L.acidophilus *%								0.209
Combination vs BMI								0.002
Combination vs *L.acidophilus *%								0.035

**Figure 6:**
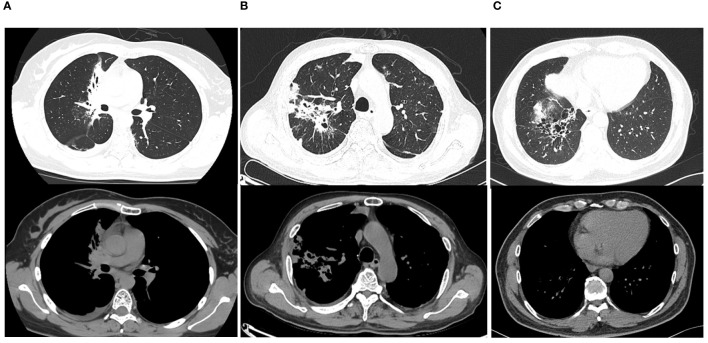
Three typical cases presenting with diffuse lung parenchymal lesions on lung imaging are shown. Case 1 **(A)**: A female patient, 32 years old, with a diffuse parenchymal lesion in the middle lobe of the right lung, in which bronchiectasis with enlarged mediastinal hilar lymph nodes and pleural effusion on the right side was seen, was finally diagnosed with adenocarcinoma of the right lung; Case 2 **(B)**: A male patient, 53 years old, with a diffuse parenchymal lesion in the upper lobe of the right lung, with uneven internal density, visible multiple cystic translucent areas, and multiple small patchy shadows in both lungs, accompanied by pleural effusion on the right side of the chest, was finally diagnosed as a lung infection caused by *Klebsiella. Pneumonia*; Case 3 **(C)**: A male patient, 52 years old, with diffuse parenchymal lesions in the lower lobe of the right lung, and multiple cystic translucent areas of varying sizes with peripheral striated shadows, was finally diagnosed with congenital airway malformation.

We used data on the BMI and *L. acidophilus* % in BALF of three typical cases showing Diffuse lung parenchymal lesions on CT imaging who had final diagnoses of lung cancer (**Figure 6A**), lung infection (**Figure 6B**), and congenital lung malformation (**Figure 6C**) and input these into the above prediction equation. The results showed that the probability of lung cancer in these three patients was 98.61%, 0.10%, and 0.09%, respectively.

### The microbial interactions in the LRT of patients with lung cancer or lung infections are weakened

**Figure 7:**
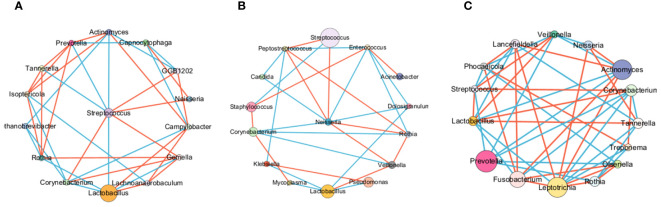
The LRT microbiome interactions with each other in different groups. BALF microbiome networks in lung cancer **(A)**, lung infection**(B)**, and others **(C)**. The networks of the top 15 genera were built by SparCC (Sparse Correlations for Compositional data) for different diseases. Each node represents a genus. The size of the nodes represents the relative abundance of the genus. Each edge represents a significant correlation between pairs of nodes (P<0.05). The width of the edge is proportional to the absolute correlation coefficient. Edges were colored based on co-existence (red) or co-exclusion (blue) relationship. The network was drawn using Cytoscape software.

To explore potential microbiome coexistence and coexclusion relationships, we performed a co-occurrence network analysis. We selected the top 15 most abundant genera in each group and used them to build and estimate a network based on the relative abundances of microbiome genera using SparCC (Sparse Correlations for Compositional data) with an r threshold = 0.7 and a p threshold = 0.01. The degree of connectivity between genera was weaker in the lung cancer group. *Lactobacillus*, the most abundant genus, had few connections with other genera, while *Streptococcus*, the second most abundant genus, had connections with the other eight genera (**Figure 7A**). The degree of connectivity between genera was also weaker in the lung infection group, with *Neisseria* showing more connections to other genera. (**Figure 7B**). The genera showed a strong degree of connectivity in the others group, with *Prevotella* being mutually exclusive with the other six genera (**Figure 7C**). This indicated the weakened interaction between microbes in the LRT of patients with lung cancer or lung infections. The numbers presented in **Tables 8**–**10** represent the correlation coefficient using SparCC for the top 15 genera in BALF across the three groups.

**Table 8:** The correlation coefficient by SparCC of the top 15 genera in BALF for the lung cancer group.

Variable	*Actinomyces*	*Campylobacter*	*Capnocytophaga*	*Corynebacterium*	*Gemella*	*GGB1202*	*Isoptericola*	*Lachnoanaerobaculum*	*Lactobacillus*	*Methanobrevibacter*	*Neisseria*	*Prevotella*	*Rothia*	*Streptococcus*	*Tannerella*
*Actinomyces*	1														
*Campylobacter*	0.116	1													
*Capnocytophaga*	0.013	-0.141	1												
*Corynebacterium*	-0.088	-0.174	-0.028	1											
*Gemella*	-0.068	-0.042	-0.032	0.316	1										
*GGB1202*	-0.221	0.186	-0.151	0.084	0.049	1									
*Isoptericola*	0.241	-0.023	-0.019	-0.165	-0.025	-0.078	1								
*Lachnoanaerobaculum*	-0.057	0.109	-0.061	0.043	0.147	-0.014	-0.165	1							
*Lactobacillus*	0.019	0.352	0.016	-0.106	-0.153	0.028	0.031	0.107	1						
*Methanobrevibacter*	-0.163	-0.015	0.078	-0.113	0.062	0.041	-0.023	0.000	0.091	1					
*Neisseria*	0.023	0.082	0.118	-0.162	-0.036	-0.062	-0.098	0.092	-0.06	-0.072	1				
*Prevotella*	0.211	-0.157	-0.134	0.054	-0.071	-0.062	0.205	-0.012	0.003	0.024	-0.082	1			
*Rothia*	-0.025	-0.088	-0.061	0.026	0.128	-0.015	0.107	-0.077	-0.108	-0.121	-0.112	0.147	1		
*Streptococcus*	-0.146	0.016	0.205	0.239	0.289	0.105	-0.188	0.028	-0.552	0.041	0.202	-0.164	-0.069	1	
*Tannerella*	0.407	-0.086	0.036	-0.029	-0.13	-0.067	0.268	-0.01	0.074	0.011	0.039	0.351	0.138	-0.265	1

**Table 9:** The correlation coefficient by SparCC of the top 15 genera in BALF for the lung infection group.

Variable	*Streptococcus*	*Lactobacillus*	*Pseudomonas*	*Staphylococcus*	*Corynebacterium*	*Veillonella*	*Acinetobacter*	*Neisseria*	*Rothia*	*Klebsiella*	*Enterococcus*	*Candida*	*Peptostreptococcus*	*Dolosigranulum*	*Mycoplasma*
*Streptococcus*	1.000														
*Lactobacillus*	0.130	1.000													
*Pseudomonas*	-0.085	-0.119	1.000												
*Staphylococcus*	0.044	-0.029	-0.042	1.000											
*Corynebacterium*	-0.088	-0.035	0.074	0.033	1.000										
*Veillonella*	0.352	0.065	-0.043	-0.068	-0.030	1.000									
*Acinetobacter*	-0.034	-0.038	0.121	0.010	0.083	-0.056	1.000								
*Neisseria*	0.146	0.132	-0.023	-0.040	-0.067	0.138	-0.067	1.000							
*Rothia*	0.364	0.004	-0.058	-0.002	-0.058	0.193	-0.037	0.076	1.000						
*Klebsiella*	0.002	-0.187	0.158	0.020	-0.011	-0.029	0.023	-0.062	0.055	1.000					
*Enterococcus*	-0.094	-0.017	0.005	0.149	0.138	-0.076	0.038	-0.069	-0.036	0.041	1.000				
*Candida*	-0.060	0.082	0.028	0.045	0.010	-0.019	0.049	-0.093	-0.088	-0.042	0.023	1.000			
*Peptostreptococcus*	0.313	0.068	-0.056	-0.084	-0.007	0.271	-0.037	0.162	0.253	-0.103	-0.062	-0.107	1.000		
*Dolosigranulum*	0.022	-0.021	0.029	-0.024	0.282	0.059	-0.037	-0.088	-0.009	-0.016	-0.037	-0.032	0.105	1.000	
*Mycoplasma*	-0.049	-0.111	0.042	0.039	-0.027	0.005	0.022	-0.069	-0.053	0.042	0.051	0.111	-0.085	-0.018	1.000

**Table 10:** The correlation coefficient by SparCC of the top 15 genera in BALF for the others group.

Variable	*Actinomyces*	*Corynebacterium*	*Fusobacterium*	*Lactobacillus*	*Lancefieldella*	*Leptotrichia*	*Neisseria*	*Olsenella*	*Phocaeicola*	*Prevotella*	*Rothia*	*Streptococcus*	*Tannerella*	*Treponema*	*Veillonella*
*Actinomyces*	1														
*Corynebacterium*	-0.051	1													
*Fusobacterium*	0.408	-0.105	1												
*Lactobacillus*	-0.159	0.236	-0.221	1											
*Lancefieldella*	0.162	0.030	0.376	-0.143	1										
*Leptotrichia*	0.407	-0.222	0.697	-0.171	0.273	1									
*Neisseria*	0.111	0.003	0.018	-0.048	-0.098	0.164	1								
*Olsenella*	0.086	-0.083	0.285	-0.012	0.093	0.246	-0.044	1							
*Phocaeicola*	-0.012	0.021	0.114	-0.041	0.128	0.036	0.032	-0.139	1						
*Prevotella*	-0.304	-0.231	0.091	0.038	-0.003	-0.002	0.095	-0.125	0.097	1					
*Rothia*	0.047	-0.078	-0.016	0.019	0.008	0.132	0.044	0.189	-0.079	-0.463	1				
*Streptococcus*	0.041	0.131	-0.074	0.356	-0.228	0.038	-0.044	0.013	-0.189	-0.121	0.076	1			
*Tannerella*	-0.043	-0.103	-0.054	0.132	-0.103	-0.121	0.192	0.018	0.031	0.076	-0.115	0.035	1		
*Treponema*	0.015	0.151	-0.066	0.05	0.048	-0.107	0.013	-0.021	0.116	-0.014	-0.118	0.041	-0.094	1	
*Veillonella*	-0.135	0.025	0.093	0.341	-0.098	0.093	-0.141	0.013	-0.124	-0.131	0.074	0.221	0.059	-0.071	1

## Discussion

Lung cancer is a common tumor worldwide, and respiratory microorganisms play an important role in lung cancer development. However, there are limitations to the current diagnostic and differential diagnostic tools for lung cancer (Chinese Anti-Cancer Association et al., 2021). We analyzed the clinical characteristics and the LRT microbiome of patients with manifestations of Diffuse lung parenchymal lesions on imaging. The microbial diversity was significantly diminished, and the relative abundance of *L. acidophilus* was significantly increased in the LRT of patients with lung cancer. Collectively, our findings suggest that the BMI combined with the relative abundance of *L. acidophilus* in the BALF is a good predictor of lung cancer risk.

We analyzed the microbiome of BALF by metagenomic sequencing. The diversity of the LRT microbiota of patients with lung cancer was diminished. A large-scale LRT microbiome study conducted by Yu et al (Yu et al., 2016). also reported that the microbiota richness was significantly lower in patients with lung cancer, with a gradual decrease in diversity from healthy to non-cancerous to cancerous sites. Hosgood et al (Hosgood et al., 2021). sequenced mouthwash samples of patients with lung cancer and controls, revealing that those with a lower microbiota alpha diversity had a significantly increased risk of lung cancer.

The development of high-throughput sequencing technologies has led to an increasing number of studies reporting diverse microbial flora in the lungs, as well as significant differences between physiological and pathological states (Baranova et al., 2022). Although the lung microbiota is constantly updated and replaced, most microbes belong to *Bacteroidetes*, Firmicutes, *Proteobacteria*, and *Actinobacteria*. The main genera found in the lungs of healthy people include *Prevotella*, *Veillonella*, *Streptococcus*, *Neisseria*, *Haemophilus*, and *Fusobacterium* (Kovaleva et al., 2019). Our study revealed changes in the LRT bacterial flora of patients with lung cancer, showing a significant enrichment of *Lactobacillus* (which belongs to Firmicutes). These findings are consistent with Hosgood et al (Hosgood et al., 2021), who reported that an association between a higher abundance of *Lactobacillus* and increased lung cancer risk. These studies have established robust associations between lung cancer and specific microorganisms, such as *Haemophilus influenza, Acidovorax, Klebsiella, Moraxella catarrhalis, Mycobacterium tuberculosis, and Granulicatella adiacens* (Chen et al., 2021).

In contrast, Lee et al (Lee et al., 2016). analyzed BALF from 20 patients with lung cancer and showed that compared with healthy people, the relative abundance of two phyla (Firmicutes and Saccharibacteria) and two genera (*Veillonella* and *Megasphaera*) was higher in patients with lung cancer. Furthermore, Liu et al (Liu et al., 2018). reported a significant increase in the relative abundance of *Streptococcus* in BALF of patients with lung cancer. In summary, these studies suggest that the LRT microbiota undergoes dynamic changes during lung cancer development and that microecological imbalance occurs in patients with lung cancer.

Our findings suggest the BMI combined with the relative abundance of *L. acidophilus* in the BALF may be a good predictor of lung cancer risk. A lower BMI was associated with a higher risk of lung cancer, which is consistent with studies by Smith et al (Smith et al., 2012). and Yu et al (Yu et al., 2018). *L. acidophilus* is a homofermentative, microaerobic, short-chain Gram-positive bacterium (Anjum et al., 2014). It maintains thermal stability and activity over a wide pH range and is a strong inhibitor of food spoilage and pathogenic bacteria, making it an important class of biological preservatives (Vemuri et al., 2018). *L. acidophilus* is generally considered a probiotic in the medical field, and its immunomodulatory ability has been demonstrated *in vitro* (Vemuri et al., 2018). Furthermore, it modulates microbiota and reduces inflammation levels in clinical models (Martoni et al., 2020). In terms of disease treatment, *L. acidophilus* significantly improves the abdominal pain and symptom severity scores of adult patients with irritable bowel syndrome and normalizes bowel habits accordingly (Paul et al., 2021). Furthermore, patients with rheumatoid arthritis showed improved symptoms after consuming *L. acidophilus* preparations (Yang et al., 2020). The protective and regulatory effects of *L. acidophilus* were also reflected in a 30% reduction in lung cancer risk that was associated with the high consumption of yogurt, indicating its potential protective role in lung cancer development. Simultaneous administration of *L. acidophilus* and cisplatin reduces lung tumor size, improves survival rate, and regulates the antigrowth and proapoptotic effects of cisplatin (Gui et al., 2015). In conclusion, *L. acidophilus* plays a protective or ameliorating role in the occurrence and development of many diseases, including lung cancer. Our study revealed the significant enrichment of probiotics in the LRT of patients with lung cancer. This may be due to carcinogenesis and subsequent alteration of the lung microenvironment, thereby paradoxically promoting the proliferation of probiotics to exert anticancer effects. Needless to say, this speculation requires further verification. The mechanisms of microbiota involvement in cancer occurrence and progression remain unclear. Studies have shown that changes in the LRT microbiota may contribute to the occurrence or progression of lung cancer through mechanisms such as the inflammatory response, immune response, and metabolic product regulation (Francescone et al., 2014; Gur et al., 2015; O'Keefe et al., 2015).

Our study has several limitations. Firstly, the sample size was relatively small, there was no control group of healthy individuals and a larger validated cohort, and the majority of study participants resided in the same geographic area, which limits the generalizability of our findings. Secondly, it is regrettable that well did not extract RNA from the BALF samples for sequencing, which prevented further analysis of the functional differences of these bacteria in the human body. The role of the LRT microbiota in lung cancer tumorigenesis is largely unknown, and further study is necessary. Third, we did not consider lung function in our study. It was reported that the respiratory microbiome of patients with chronic obstructive pulmonary disease differed from that of healthy individuals (Wang et al., 2021), and this confounding factor must be addressed in a follow-up study. Finally, our study did not address daily dietary composition, which may induce alterations in the lung microbiome through the gut–lung axis (Marsland et al., 2015).

## Conclusion

Our study revealed that in patients where lung imaging shows diffuse parenchymal lesions, an imbalance in the component ratio of the microbial community, diminished microbial diversity, and the presence of specific microbial markers in the LRT microbiome can predict lung cancer risk. Our findings provide novel approaches to the diagnosis and differential diagnosis of lung cancer.

## Data Availability

The datasets presented in this study can be found in online repositories. The names of the repository/repositories and accession number(s) can be found in the article/**Supplementary Material**.

## Supplementary material

SUPPLEMENTARY FIGURE 1 Heat maps were presented to demonstrate the microbiome composition of each sample for all phyla **(A)**, the top 50 genera **(B)**, and the top 50 species **(C)**.
